# The GA-BPNN-Based Evaluation of Cultivated Land Quality in the PSR Framework Using Gaofen-1 Satellite Data

**DOI:** 10.3390/s19235127

**Published:** 2019-11-22

**Authors:** Shanshan Liu, Yiping Peng, Ziqing Xia, Yueming Hu, Guangxing Wang, A-Xing Zhu, Zhenhua Liu

**Affiliations:** 1 College of Natural Resources and Environment, South China Agricultural University, Guangzhou 510642, China; Shanl633@163.com (S.L.); pyp_scau@163.com (Y.P.); xzq@stu.scau.edu.cn (Z.X.); gxwang@siu.edu (G.W.); azhu@wisc.edu (A.-X.Z.); 2 Guangdong Provincial Key Laboratory of Land Use and Consolidation, South China Agricultural University, Guangzhou 510642, China; 3 Guangdong Province Engineering Research Center for Land Information Technology, South China Agricultural University, Guangzhou 510642, China; 4 Key Laboratory of Construction Land Transformation, Ministry of Land and Resources, South China Agricultural University, Guangzhou 510642, China; 5 Department of Geography and Environmental Resources, College of Liberal Arts, Southern Illinois University Carbondale (SIUC), Carbondale, IL 62901, USA; 6 Department of Geography, University of Wisconsin-Madison, Madison, WI 53706, USA

**Keywords:** cultivated land quality, spectral indices, GA-BPNN model

## Abstract

Rapid and efficient assessment of cultivated land quality (CLQ) using remote sensing technology is of great significance for protecting cultivated land. However, it is difficult to obtain accurate CLQ estimates using the current satellite-driven approaches in the pressure-state-response (PSR) framework, owing to the limitations of linear models and CLQ spectral indices. In order to improve the estimation accuracy of CLQ, this study used four evaluation models (the traditional linear model; partial least squares regression, PLSR; back propagation neural network, BPNN; and BPNN with genetic algorithm optimization, GA-BPNN) to evaluate CLQ for determining the accurate evaluation model. In addition, the optimal satellite-derived indicator in the land state index was selected among five vegetation indices (the normalized vegetation index, NDVI; enhanced vegetation index, EVI; modified soil-adjusted vegetation index, MSAVI; perpendicular vegetation index, PVI; and soil-adjusted vegetation index, SAVI) to improve the prediction accuracy of CLQ. This study was conducted in Conghua District of Guangzhou, Guangdong Province, China, based on Gaofen-1 (GF-1) data. The prediction accuracies from the traditional linear model, PLSR, BPNN, and GA-BPNN were compared using observations. The results demonstrated that (1) compared with other models (the traditional linear model: R^2^ = 0.14 and RMSE = 91.53; PLSR: R^2^ = 0.33 and RMSE = 74.58; BPNN: R^2^ = 0.50 and RMSE = 61.75), the GA-BPNN model based on EVI in the land state index provided the most accurate estimates of CLQ, with the R^2^ of 0.59 and root mean square error (RMSE) of 56.87, indicating a nonlinear relationship between CLQ and the prediction indicator; and (2) the GA-BPNN-based evaluation approach of CLQ in the PSR framework was driven to map CLQ of the study area using the GF-1 data, leading to an RMSE of 61.44 at the regional scale, implying that the GA-BPNN-based evaluation approach has the potential to map CLQ over large areas. This study provides an important reference for the high-accuracy prediction of CLQ based on remote sensing technology.

## 1. Introduction

Cultivated land is the most basic capital good of agricultural production [1,2]. The area of cultivated land in China covers 134.9 × 10^6^ hm^2^, accounting for 19.71% of the land area. In recent years, the rapid urbanization and industrialization in China have resulted in a strong demand for evaluating cultivated land quality (CLQ) to ensure national food security. The evaluation of CLQ represents natural conditions and the degree of anthropogenic use of cultivated land resources, involving the quantification and grading of CLQ indices [3,4,5]. Therefore, it is very important to quickly and effectively evaluate CLQ. However, traditional estimations of CLQ are based on field measurements, which are time-consuming and costly [6]. Traditional methods are also unable to output high precision spatial distributions of CLQ. Remote sensing technology provides a unique means for the rapid evaluation of CLQ in a time-efficient and low-cost manner. Substantial research has been conducted for the evaluation of CLQ based on remote sensing data [7,8,9,10]. 

The current research on remote sensing-based CLQ evaluation methods was divided into two categories: CLQ quasi-quantity evaluation and CLQ quantity evaluation. In CLQ quasi-quantity evaluation, remote sensing data were used for the inversion of traditional some indicators (e.g., soil fertility). For example, Yu (2012) constructed an evaluation indicator system with the normalized vegetation index (NDVI) extracted from MODIS, soil organic matter (SOM), and soil mass and trace elements content obtained from field sampling to evaluate CLQ [11]. Yang et al. (2012) used Landsat TM5 to invert soil texture and SOM, and combined these with traditional indicators (barrier depth and profile configuration) to evaluate CLQ [12]. However, the CLQ quasi-quantity evaluation method is not able to increase the evaluation efficiency owing to the limitations of the traditional field measurements.

With the development of the remote sensing technique, we get CLQ quantity evaluation, whereby remote sensing data were used to evaluate CLQ based on the pressure-state-response (PSR) framework in terms of resistance to pressure, current state, and land use response [7,13]. For example, Fang et al. (2008) used Slope, ratio vegetation index (RVI), NDVI, DVI, and land use degree (LUD) to establish an evaluation model for CLQ, while Liu et al. (2010) used slope, sandy area ratio in a pixel (SARP), modified soil-adjusted vegetation index (MSAVI), soil and vegetation moisture index (SVMI), and LUD to establish a linear evaluation model of CLQ [8,9]. Although these studies have demonstrated rapid CLQ evaluation processes, they estimate and map CLQ based on the PSR framework using the remote sensing technique, in which linear relationships of CLQ with spectral indices are often assumed, and there is a lack of optimizing selection of spectral indices. Thus, it is difficult to obtain accurate CLQ estimates owing to the limitations of linear models and CLQ spectral indices.

The objective of this study was to improve the evaluation accuracy of the satellite-driven approach in the PSR framework by determining the optimal evaluation models among the traditional linear model, partial least squares regression (PLSR), back propagation neural network (BPNN), and BPNN with genetic algorithm (GA-BPNN), and selecting the evaluation indicator in the land state index by analyzing the correlation coefficient between five vegetation indices (VIs) derived from Gaofen-1 (GF-1) data and soil sample fertility (SOM; and total nitrogen, TN). This study was validated using GF-1 data in the Conghua District of Guangzhou, Guangdong Province, China. It is expected that the modifications can increase the accuracy of CLQ estimates.

## 2. Materials and Methods

### 2.1. Study Area 

This study area is Conghua District of Guangzhou City, China (113 17′–114 04′ E, 23 22′–23 56′ N), located in the transition zone from the Pearl River Delta to the northern mountain area of Guangzhou. This is a typical hilly and mountainous area and is dominated by the south subtropical monsoon climate, with an annual average temperature of 19.5–21.2 °C and an annual average precipitation of 2176.3 mm. The area of cultivated land in Guangzhou covers 13,485.99 hm^2^, of which the paddy field was 11,885.16 hm^2^, accounting for 87.91% of the total area. As the main crop, rice can be planted in two seasons.

### 2.2. Sample Data

**Figure 1:**
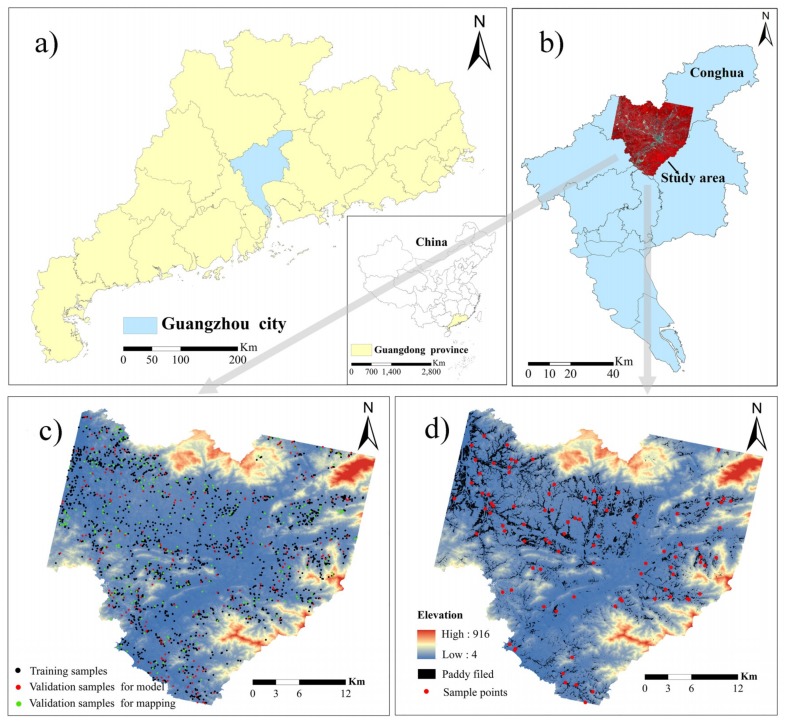
Study area and sampling distribution: (**a**) the study area location in Guangzhou City; (**b**) the standard pseudo-color map of the study area in Conghua District; (**c**) the spatial distribution of 2000 cultivated land quality (CLQ) samples (the training sample plots in black, the validation sample plots for model in red, and the validation sample plots for mapping in green); and (**d**) soil samples for selection of soil fertility indicator.

A total of 2000 samples within Conghua District were collected at a plot-scale sampling grid according to a stratified sampling method (Figure 1c). CLQ data from the 2000 samples were obtained from the CLQ map for 2016 in the Conghua National Land Department. Further, the CLQ data calculated using gradation regulations on agriculture land quality in China (GB/T 28407-2012) were considered as standard reference values of CLQ. Out of the 2000 samples, 1500 samples in black were used to the establish evaluation models, 250 sample plots in red were employed to validate the accuracy of the estimated CLQ, and another dataset of 250 sample plots in green was used to assess the accuracy of mapping CLQ at the regional scale.

In order to determine the optimal soil fertility indicator, the layout of 89 soil samples was extracted from the 2000 samples based on soil type and land-use (Figure 1d). The 89 soil samples (at 0–20 cm depth) were collected via GPS positioning in May 2016. Within each of the sample plots, soil samples were collected at five points. Each soil sample was composed by mixing subsamples from five points. One of the five points was located at the plot center and the remaining four points were allocated along the diagonal lines of the plot, with an equal distance between the points. The samples were air-dried naturally in the laboratory, and gravel and animal and plant residues were removed. The soil fertility parameters of SOM and TN contents were analyzed. SOM content was determined by the potassium dichromate oxidation-external heating method, and TN was determined using the phenol disulfonic acid colorimetric method [14].

### 2.3. Satellite Image Data and Preprocessing

A GF-1 multi-spectral image of October 2016 with a spatial resolution of 8 m was obtained from China Center for Resources Satellite Data and Application (http://218.247.138.119:7777/DSSPlatform/index.html). A Landsat-8 thermal infrared sensor (TIRS) image and a digital elevation model (DEM) from October 2016 with a spatial resolution of 30 m were obtained from the Geospatial Data Cloud of the Computer Network Information Center, Chinese Academy of Sciences (http://www.gscloud.cn). 

The radiance calibration of the GF-1 and Landsat-8 TRIS images was to convert the *DN* value of the raw image to surface spectral reflectance, which was performed based on Equation (1), expressed as follows:

(1) L=Gain×DN+Bias,

where L is the radiance, and Gain and Bias are the calibration coefficients. The values of the radiance calibration parameters are reported in Table 1. The GF-1 and Landsat-8 TRIS data were conducted using the FLAASH model. Geometric corrections and resampling were performed for the DEM and Landsat 8 TRIS imagery using ENVI 5.3. The geometric precision correction was performed on them using a quadratic polynomial calculation model. The calibration error was within 0.5 pixels. 

### 2.4. Selecting Satellite-Derived Indicators for CLQ Evaluation

CLQ evaluation indicators were obtained from the land pressure resistance index (PRI), land state index (LSI), and land use response index (LURI) based on the PSR proposed by Dumanski and Pieri [15], shown in Table 2. 

At the satellite-derived indicator layer, each indicator was selected based on the project layer (PRI, LSI, and LURI). The PRI represents the ability to withstand environmental pressure and the probability of degradation. As PRI increases, the ability to withstand environmental pressure improves and the degradation probability falls. The environmental pressure within the study area mainly originates from soil erosion. Therefore, slope was selected as the PRI evaluation indicator (Table 2). In addition, LSI denotes the current quality and potential productivity of cultivated land, reflected by soil moisture and soil fertility. Furthermore, soil organic matter (SOM) and total nitrogen (TN) are the major determinants and indicators of soil fertility [16,17]. The temperature vegetation drought index (TVDI) is related to surface soil moisture owing to changes in thermal inertia and evaporative control (evaporation and transpiration) on net radiation partitioning (energy balance) [18]. Thus, soil moisture was replaced by TVDI in this study (Table 2), expressed as follows [19]:

(2) $$TVDI=\frac{LST-LST_{min}}{a+bNDVI-LST_{min}},$$

where $LST_{min}$ is the minimum surface temperature, defining the wet edge; *LST* is the observed surface temperature at the given pixel; NDVI is the observed normalized difference vegetation index; and a and b are parameters defining the dry edge, which is modeled as a linear fit to the data ($LST_{max}=\;a+b\times NDVI$), where $LST_{max}$ is the maximum surface temperature observation for a given NDVI. More specifically, a = 48.80 and b = −33.15. TVDI is lower for wet and higher for dry conditions, with values of 1 at the dry edge and 0 at the wet edge [19].

Soil fertility (SOM and TN) is reflective of vegetation growing conditions [8,20]. Thus, remote sensing VIs (Table 2 and Table 3) were selected as soil fertility indicators in the study. In order to improve the accuracy of the CLQ evaluation, the Pearson correlation was used to select the optimal satellite-derived VI as soil fertility indicators. In particular, those with the greatest correlation coefficients between soil fertility and satellite-derived VIs under the significance level of *p* ≤ 0.05 were chosen. The Pearson correlation coefficient was derived, expressed as follows:

(3) $$r_{i}=\frac{\sum_{n=1}^{N}\left(R_{ni}-\bar{R_{i}}\right)\left(y-\bar{y}\right)}{\sqrt{\sum_{n=1}^{N}{\left(R_{ni}-\bar{R_{i}}\right)}^{2}\sum_{n=1}^{N}{\left(y-\bar{y}\right)}^{2}}},$$

where $r_{i}$ is the correlation coefficient between soil fertility and a satellite-derived VI, $R_{ni}$ is the satellite-derived VI of the nth soil sample, $\bar{R_{i}}$ is the mean value of the VI in the ith soil sample, y is the *n*th soil fertility, and $\bar{y}$ is the average value of soil fertility.

In the study area, land use embodied mainly in the form of land use types, road accessibility (RA), and land patch fragmentation are the farmers’ response to CLQ [28,29,30,31]. As paddy fields are the only type of cultivated land in this study, RA (Table 2) and land patch fragmentation were selected as LURIs indicators. RA was calculated according to Equation (4):

(4) $$\mathrm{RA}=1-\frac{d_{i}}{d},$$

where $d_{i}$ is the proximity distance between the ith land patch and road and d is the maximum distance among all land patches and the road. RA ϵ (0, 1), where larger RA values indicate higher plot accessibilities. The land patch fragmentation degree is reflected by the patch fractal dimension (PFD) (Table 2), expressed as follows:

(5) $$\mathrm{PFD}=2\times \ln\left(\frac{L}{4}\right)/\ln A,$$

where L (m) is the perimeter of the land patch and A (m^2^) is the area of land patch. PFD ϵ (1, 2), with values approaching 1 indicate a greater degree of cultivated land utilization by farmers. 

### 2.5. Modeling and Mapping Methods

In order to improve the CLQ evaluation accuracy, linear (PLSR) and nonlinear models (BPNN and GA-BPNN) were applied for CLQ predictions using satellite-derived indicators.

#### 2.5.1. Linear Model 

The traditional linear model [7,8,9] was constructed taking measured CLQ in 1500 training samples as the dependent variable and the corresponding evaluation indicator as independent variables.

(6) $$Y=\overset{n}{\underset{i=1}{\sum }}\beta_{i}{\overset{ˇ}{X}}_{i},$$

where Y is the dependent variable (CLQ value), ${\overset{ˇ}{X}}_{i}$ is the ith independent variable (evaluation indicator value), and $\beta_{i}$ is the ith weight coefficient determined from expert experience. In this study, a score within the range of [0, 3000] was assigned to CLQ with reference to the GB/T 28407-2012. Therefore, all indicators or indices were normalized to a range of 0–3000.

The PLSR is a standard multivariate statistical technique that was originally developed by Herman Wold in 1966 [32]. Previous studies [33] indicate that the PLSR is highly effective in different disciplines because it allows for the analysis of data with strong correlations in the predictor variables. The PLSR used in the study is expressed as follows:

(7) Y=Xβ+ε,

where Y is the dependent variable (CLQ value), X is the independent variable (evaluation indicator value), β is the coefficient matrix, and ε is the residual matrix.

#### 2.5.2. Back Propagation Neural Network 

BPNN is a multi-layer feed forward network trained by the error back propagation algorithm, and is suitable for various types of nonlinear relationship analysis methods. It consists of an input layer, an output layer, and several hidden layers [34,35]. *Trainlm* and *Purelin* were selected as the training function and the transfer function of the output layer in the BPNN, respectively. The steepest descent method and the back-propagation algorithm in the BPNN model were used to repeatedly adjust the weight and deviation of the network until the actual value and the expected output were as close as possible [36,37].

The hidden layer information acquired from the input layer was expressed as follows:

(8) $$o_{j}=f_{i}\left(\sum \omega_{ji}o_{i}+\theta_{j}\right),$$

where $o_{i}$ is the input layer information (the evaluation indicator); $o_{j}$ is the hidden layer information; $\omega_{ji}$ is the weight of the input layer to the hidden layer; and $f_{i}$ is the transfer function of the input layer to the hidden layer. In this study, the Trainlm function is chosen and $\theta_{j}$ is the hidden layer threshold.

The hidden layer was transferred to the output layer, expressed as follows:

(9) $$o_{k}=f_{j}\left(\sum \omega_{kj}o_{j}+\theta_{k}\right),$$

where $o_{k}$ is the output layer information (CLQ), $f_{j}$ is the transfer function of the hidden layer to the output layer (the Purelin function was used in this study), $\omega_{kj}$ represents the weight of the hidden layer to the output layer, and $\theta_{k}$ is the output layer threshold.

The number of neurons in the hidden layer was determined according to the empirical formula in Equation (9):

(10) $$n_{h}=2n_{i}+1,$$

where $n_{h}$ is the number of hidden layer units and $n_{i}$ is the number of input units.

If the estimated value differs greatly from the measured value, it is transferred to the error of the back propagation process. The process of reverse propagation uses the Levenberg–Marquardt algorithm from the output layer to the input layer to modify the connection weight in order to reduce the mean square error (MSE), expressed as follows:

(11) $$\mathrm{MSE}=\frac{1}{N}\sum {\left(o-o_{k}\right)}^{2},$$

where o is the measured CLQ, $o_{k}$ is the estimated CLQ, and N is the number of training samples.

#### 2.5.3. Genetic Algorithm-Back Propagation Neural Network

In order to improve the prediction accuracy of the BP algorithm, GA was introduced to the BP model to optimize the weight and threshold selection of the neural network. GA has the advantages of only requiring fitting information and not tending to a local solution [38,39]. Thus, the combined GA-BPNN model was used to estimate CLQ in this study. The original BPNN weight and threshold were converted into chromosomes in GA using real-number coding. The code length was calculated using Equation (11):

(12) S=i*j+j*k+j+k,

where i is the number of input layer neuron nodes, which is the number of evaluation indicators; j is the number of neurons in the hidden layer; and k is the number of neurons in the output layer. Note that, in this case, the output layer only exhibited CLQ, hence k=1. This was followed by the generation of a random population of chromosomes. The BPNN was used to obtain the individual fitness value (E), which is the sum of the absolute error between the estimated and measured values of the training data, expressed as follows:

(13) $$min\;E=\sum abs\left(y_{k}-o_{k}\right),$$

where $y_{k}$ is the measured value of CLQ in the *k*th land patch and $o_{k}$ is the estimated CLQ value in the *k*th land patch.

## 3. Results

### 3.1. Satellite-Derived Indicators of CLQ Evaluation

**Figure 2:**
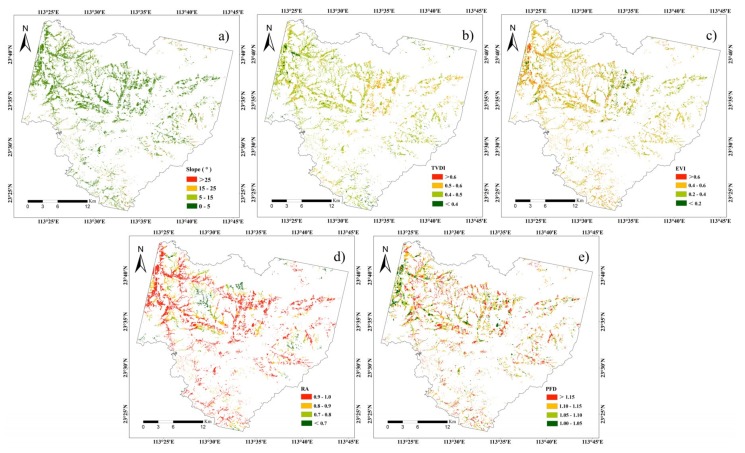
Spatial distributions of satellite-derived indicators in the pressure-state-response (PSR) framework: (**a**) slope; (**b**) temperature vegetation drought index (TVDI); (**c**) enhanced vegetation index (EVI); (**d**) road accessibility (RA); and (**e**) patch fractal dimension (PFD).

The satellite-derived indicators in the three project layers were calculated in the PSR framework. The PRI slope was determined using the ArcGIS 10.2 surface analysis model, based on the DEM data (Figure 2a). Next, TVDI, acting as a satellite indicator for LSI, was derived according to Equation (2). The spatial distribution of TVDI is shown in Figure 2b. In order to acquire highly-accurate CLQ estimates, the optimal VI was selected among five VIs (NDVI, EVI, MSAVI, SAVI, and PVI) as the soil fertility indicator. In particular, the VIs with the greatest correlation coefficients with soil fertility (*p* ≤ 0.05) were chosen, as shown in Table 4. It can be seen that EVI exhibited the greatest correlation coefficients with SOM and TN (0.88 and 0.90, respectively), indicating EVI as the optimal indicator for the evaluation of CLQ. The spatial distribution of EVI is presented in Figure 2c.

Finally, RA and PFD, calculated according to Equations (4) and (5), respectively, and their spatial distributions are given in Figure 2d,e.

From the spatial distribution of spectral indicators, the slope, PFD, and TVDI with low values mainly located in the northwest part of the study area, and EVI and RA with high values also located in this part. This indicates that, the larger the EVI and RA, the smaller the slope, PFD, and TVDI, which corresponds to the factual conditions.

### 3.2. The Optimal Model for CLQ Evaluation

The selected evaluation indicators (slope, TVDI, EVI, RA, and PFD) were used as independent variables and CLQ acted as the dependent variable. The CLQ predictions of the traditional linear, PLSR, BPNN, and GA-BPNN models were compared. In order to evaluate the accuracy of the prediction models for estimating CLQ, the coefficient of determination (R^2^), root mean square error (RMSE) between the estimated and observed values, and normalized root mean square error (NRMSE) [13] were calculated based on the training and validation datasets. The obtained traditional linear and PLSR evaluation model are expressed as follows:

(14) $$\hat{Y}=\frac{1}{3}\times \overset{﹀}{Slope}+\frac{1}{3}\times \left(\frac{\overset{﹀}{EVI}+\overset{﹀}{TVDI}}{2}\right)+\frac{1}{3}\times \left(\frac{\overset{﹀}{RA}+\overset{﹀}{PFD}}{2}\right)\;\;\;\;\;\left(R^{2}=0.14,P=0\right),$$

(15) $$\hat{Y}=1480.15-2.47\times slope+569.68\times EVI-296.41\times TVDI+106.70\times RA-79.28\times PFD\;\;\;\;\;\;\;\;\;\;\;\;\;\;\;\;\;\;\;\left(R^{2}=0.33,P=0\right).$$

**Figure 3:**
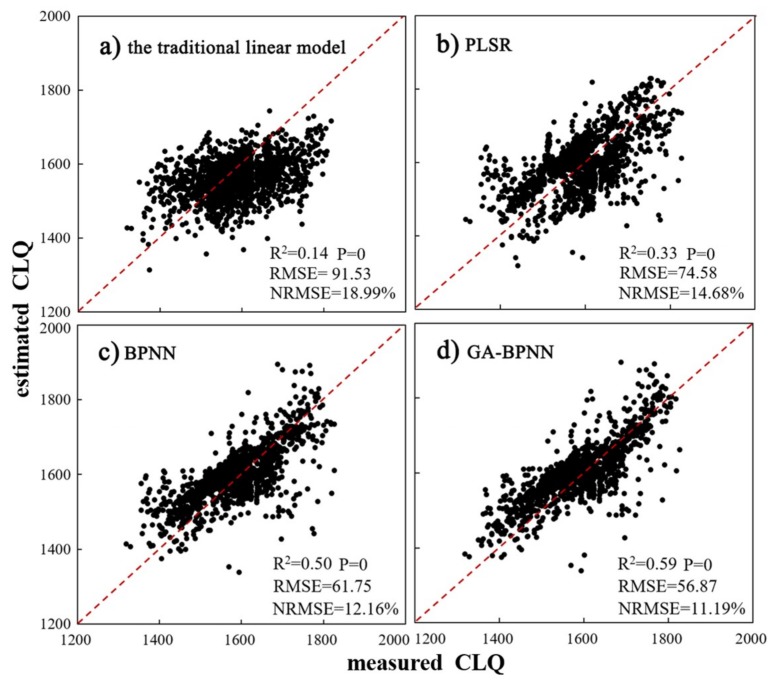
Scatterplots of measured versus estimated CLQ obtained by four models using the training dataset: (**a**) the traditional linear model; (**b**) partial least squares regression (PLSR); (**c**) back propagation neural network (BPNN), and (**d**) BPNN with genetic algorithm optimization (GA-BPNN). RMSE, root mean square error; NRMSE, normalized RMSE.

Moreover, a three-layer BPNN with a single hidden layer was used for the CLQ predictions. The number of neuron nodes in the hidden layer was fixed at 13, the number of iterations at 1000, and both the learning rate and learning objective at 0.01. In order to compare the results of the GA optimization, the network structure and parameter configuration were consistent with those of the BPNN. In the GA, the number of the maximum runs was set at 50, and the population size, crossover probability (P_c_), and mutation probability (P_m_) were fixed as 128, 0.9, and 0.02, respectively. A total of 1500 training samples were selected for the PLSR, BPNN, and GA-BPNN models in order to train the response relationships between CLQ and the selected evaluation indicators (Figure 3).

From the results using the training dataset, the explanatory power of CLQ was observed to vary greatly depending on the evaluation model (R^2^: 0.14 for traditional linear model, 0.33 for PLSR, 0.50 for BPNN, and 0.59 for GA-BPNN). At a given CLQ, the traditional linear model estimations exhibited the greatest RSME values, and GA-BPNN exhibited the lowest. The PLSR and traditional linear models performed the worst, while the BPNN and GA-BPNN models were able to improve the overall CLQ prediction accuracy. In particular, the GA-BPNN model exhibited the highest accuracy and the strongest prediction ability, implying that there existed a significant nonlinear relationship between the evaluation indicators and CLQ.

**Figure 4:**
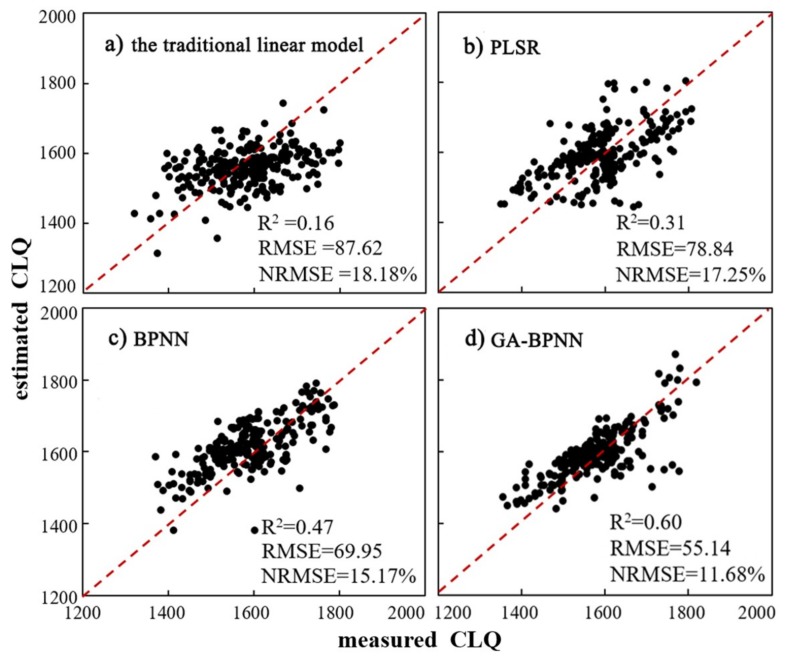
Scatterplots of measured versus estimated values of CLQ obtained four models using the 250-sample validation dataset for model: (**a**) the traditional linear model; (**b**) PLSR; (**c**) BPNN; and (**d**) GA-BPNN.

Moreover, 250 samples were used to validate the estimation accuracy of the four models, with the results presented in Figure 4. For the validation samples, the estimated and measured values of CLQ were observed to be randomly distributed on both sides of the 1:1 line, depending on the model. More specifically, for the GA-BPNN model, the scatter points were closely located to the 1:1 line, with limited outliers. Thus, the results demonstrate that the indicators selected in this study are suitable for predicting CLQ in the study area.

Furthermore, for the validation dataset, the observed prediction accuracies of the four models from high to low are GA-BPNN (R^2^ = 0.60, RMSE = 55.41) > BPNN (R^2^ = 0.47, RMSE = 69.95) > PLSR (R^2^ = 0.31, RMSE = 78.84) > traditional linear model (R^2^ = 0.16, RMSE = 87.62). This implies that the GA-BPNN model demonstrated the highest accuracy and the strongest prediction ability, consistent with the results of the training samples.

### 3.3. Spatial Prediction of Cultivated Land Quality at the Regional Scale

**Figure 5:**
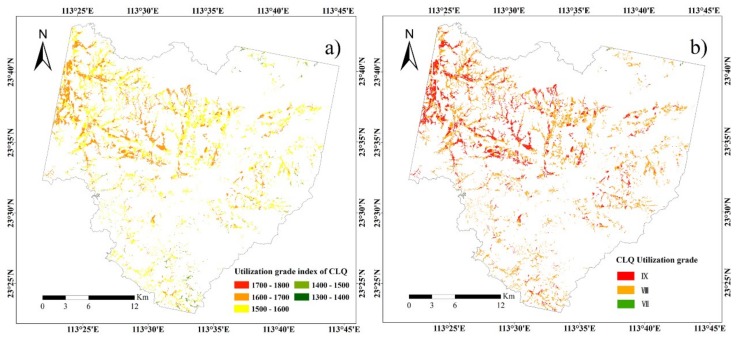
Spatial distributions of CLQ using GA-BPNN for the study area: (**a**) utilization grade index and (**b**) utilization grade.

The GA-BPNN model was applied to map CLQ in Conghua District at the regional scale using the evaluation indicators. The CLQ values were then divided into three grades according to gradation regulations on agriculture land quality in China (GB/T 28407-2012). The derived spatial distribution of CLQ was presented in Figure 5. 

**Figure 6:**
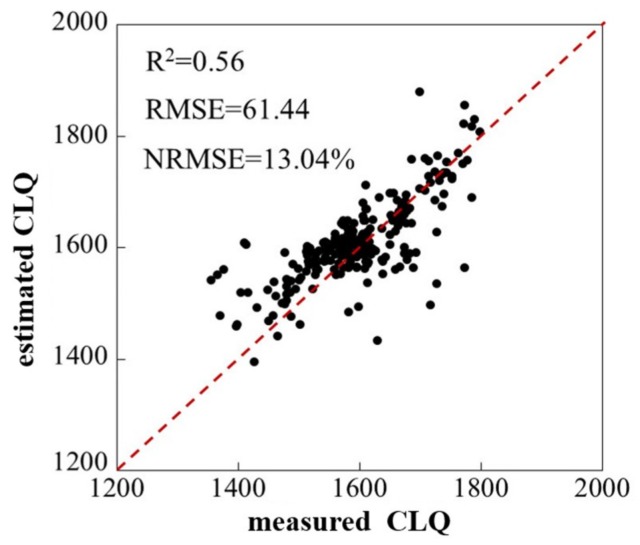
Measured and estimated CLQ based on the GA-BPNN model using the 250 validation sample plots for mapping.

In order to validate the mapping accuracy of CLQ using the GA-BPNN model, the CLQ estimated using the evaluation indicators was compared with the measured data from the 250 samples by calculating the R^2^, RMSE, and NRMSE (Figure 6). There was an improvement in the CLQ mapping using the satellite-derived indicators based on the GA-BPNN model (R^2^ = 0.56, RMSE = 61.44, NRMSE = 13.04%), indicating that the method proposed in this study is capable of estimating CLQ at the regional scale.

## 4. Discussion

The CLQ plays a vital role in agricultural production. CLQ will change drastically over a short time when environmental change and human economic activities occur. Therefore, timely assessment and dynamic monitoring of CLQ are especially critical in the agricultural vulnerable region. Estimating CLQ using remote sensing data is a cost-efficient, yet challenging method owing to the complex nonlinear relationship between CLQ and the evaluation indices [40]. Previous studies [7,8,9] based on the PSR framework using remote sensing technique have generally focused on building linear models with coefficients determined from expert experience. In order to improve the CLQ evaluation accuracy from previous work, this study applied linear (traditional linear model and PLSR) and nonlinear models (BPNN and GA-BPNN) to evaluate CLQ. 

Compared with the evaluation results from the four models, BPNN and GA-BPNN models (R^2^ of 0.50 and 0.59) performed better than PLSR and the traditional linear models (R^2^ of 0.33 and 0.14) in accurately predicting CLQ, highlighting the evident nonlinear relationships between satellite-derived indicators and CLQ. Meanwhile, this demonstrates that the GA-BPNN model performed better on CLQ evaluation than the BPNN model, because GA was introduced to BPNN, which led to an integrated GA-BPNN method to optimize the BPNN initial input parameters (thresholds and weights) and provide the solution for the problem of being stuck in the local minima [41]. The results of the validation datasets also showed that, compared with the BPNN model without the optimization of the input parameters, the GA-BPNN model significantly improved the estimation accuracies of CLQ by decreasing the RMSE values by 14.81. 

In order to further validate the regional scale applicability of the GA-BPNN prediction model, the satellite-derived indicators were used to map CLQ over Conghua District. The comparison between the measured and estimated CLQ resulted in an RMSE of 55.14 and NRMSE of 11.68%. The result demonstrated that the satellite-driven evaluation approach in the PSR framework using the GA-BPNN model has the potential to accurately map CLQ.

It has to be pointed out that the experiment was conducted only in paddy fields. Owing to the absence of information on other types of cultivated land, we cannot currently validate the evaluation indicators and CLQ model uncertainties caused by cultivated land type. Thus, more field samples from different types of cultivated land should be collected to further validate the reliability of the indicators and the model proposed in the study. Moreover, in order to further build and validate the optimal indicators of CLQ evaluations in future studies, larger sample sizes with a CLQ range from low to high should be utilized. This will improve the accuracy of the CLQ predictions. Finally, compared with the algorithm efficiency of linear models (the traditional linear and PLSR), the BPNN and GA-BPNN algorithms have the problem of slow learning, resulting in the difficulty to satisfy practical applications, especially for large-scale training samples [42]. Thus, the addition of other prediction methods (e.g., multiple linear regression, MLR; deep learning; support vector machine, SVM; and random forest, RF) is expected to enhance the efficiency and accuracy of CLQ evaluations.

## 5. Conclusions

It is agreed that estimating and mapping CLQ using satellite-derived approaches based on the PSR framework is quick and effective, yet also very challenging owing to spectral indicator accuracy, modeling methods, and model transferability, which will affect the accuracy of CLQ evaluation. In order to improve the accuracy of CLQ evaluation, this study focused on determining an accurate spectral response relationship model between CLQ and satellite-derived indicators based on the PSR framework to evaluate CLQ using the traditional linear, PLSR, BPNN, and GA-BPNN algorithms. The experiment was conducted in the Conghua district of Guangzhou City. The following conclusions could be drawn: (1) comparing with other VIs (NDVI, PVI, MSAVI, and SAVI), EVI, with the greatest correlation coefficients r of 0.88 for SOM and 0.90 for TN, is the optimal indicator in the land state index; (2) the GA-BPNN model demonstrated the strongest fitting ability for CLQ (R^2^ = 0.59 and RMSE = 56.87) compared with the PLSR, BPNN, and traditional linear models. This indicates a nonlinear relationship between CLQ and the prediction indicators; (3) the validation led to a relatively small NRMSE value of 13.04% for mapping CLQ, which further indicated that the GA-BPNN-based evaluation approach of CLQ in the PSR framework using GF-1 data was reliable to map CLQ at the regional scale. This study provides an important reference for high-accuracy CLQ predictions.

## Figures and Tables

**Table 1:** Radiance calibration parameter values of the Gaofen-1 (GF-1) satellite and Landsat-8 thermal infrared sensor (TIRS) images.

Satellite	Parameter Value	Bands				
		Band 1	Band 2	Band 3	Band 4	Band 6
GF-1	Gain	0.2072	0.1776	0.1770	0.1909	
Landsat-8 TRIS	Bias	7.5348	3.9395	−1.7445	−7.2053	
	Gain	1.1807	1.2098	0.9425	0.9692	17.04
	Bias	−7.3800	−7.6100	−5.9400	−6.0700	12.65

**Table 2:** Cultivated land quality (CLQ) evaluation indicator system. TVDI, temperature vegetation drought index; VI, vegetation index; RA, road accessibility; PFD, patch fractal dimension.

Target Layer	Project Layer	Satellite-Derived Indicator Layer
CLQ evaluation indicator system	Pressure Resistance Index (PRI)	Slope
	Land State Index (LSI)	TVDI
		VIs
	Land Use Response Index (LURI)	RA
		PFD

**Table 3:** Equations for the five VIs used in this study. NDVI, normalized vegetation index, EVI, enhanced vegetation index; SAVI, soil-adjusted vegetation index; MSAVI, modified SAVI; PVI, perpendicular vegetation index.

VIs	Algorithm Formula	Reference
NDVI	$\left(R_{NIR}-R_{RED})/(R_{NIR}+R_{RED}\right)$	[21,22]
EVI	$2.5\times (R_{NIR}-R_{RED})/(R_{NIR}+6R_{NIR}-7.5R_{BLUE}+1)$	[23]
MSAVI	$\left[2R_{NIR}+1-\sqrt{{\left(2R_{NIR}+1\right)}^{2}-8\left(R_{NIR}-R_{RED}\right)}\right]/2$	[24]
SAVI	$1.5\times (R_{NIR}-R_{RED})/(R_{NIR}+R_{RED}+0.5)$	[25]
PVI	$\left(R_{NIR}-aR_{RED}-b\right)/\sqrt{1+a^{2}}$ (a=0.9, b=0.1)	[26,27]

*R_NIR_*, *R_RED_*, and *R_BLUE_* are the spectral reflectance of near-infrared, red, and blue bands respectively.

**Table 4:** Correlation coefficients between the VIs and soil fertility parameters derived from the soil samples. SOM, soil organic matter; TN, total nitrogen.

Soil Fertility Parameters	NDVI	EVI	MSAVI	SAVI	PVI
SOM (%)	0.82 **	0.88 **	0.87 **	0.84 **	0.85 **
TN (mg/kg)	0.75 **	0.90 **	0.88 **	0.78 **	0.79 **

** correlation is significant at *p* < 0.01 level.
